# Maternal near-miss from cesarean scar ectopic pregnancy: emerging clinical challenge in resource-limited settings—a case series

**DOI:** 10.1186/s13256-026-05902-6

**Published:** 2026-03-04

**Authors:** Getasew Bayu Asnakew, Abel Gedefaw Ali, Woundu Belayneh Dejen, Abebe Melis Nisro, Shimelis Fantu Gebresilasie

**Affiliations:** 1 Department of Obstetrics and Gynecology, Hawassa University College of Medicine and Health Science, Hawassa, Ethiopia; 2 Department of Pathology, Hawassa University College of Medicine and Health Science, Hawassa, Ethiopia

**Keywords:** Cesarean scar ectopic pregnancy, Maternal near miss, Low-resource settings, Ethiopia, Case series

## Abstract

**Background:**

Cesarean scar ectopic pregnancy is a rare but increasingly recognized form of ectopic pregnancy in which a pregnancy is implanted into a myometrial defect caused by a cesarean scar. If not promptly identified, it can lead to life-threatening complications, underscoring the need for heightened clinical suspicion, timely diagnosis, and context-appropriate management. Reports from resource-limited settings remain scarce, making this case series from Ethiopia important for raising clinical awareness and highlighting the challenges of timely recognition and management.

**Case presentation:**

We reported two consecutive cases of maternal near misses that met the WHO criteria, both resulting from cesarean scar ectopic pregnancy, along with a third case involving delayed diagnosis. All were Ethiopian (East Africa), managed at a teaching hospital in Ethiopia between January 2023 and May 2025. Patient 1: a 28 year-old (gravida 4, two prior cesareans) who was initially misdiagnosed with threatened miscarriage at a gestational age of 10 weeks, was managed expectantly for 2 weeks and was later misdiagnosed with incomplete miscarriage, underwent manual vacuum aspiration, and experienced severe hemorrhage; cesarean scar ectopic pregnancy was subsequently diagnosed, ultimately requiring emergency hysterectomy and transfusion of five units of whole blood. Patient 2: a 25 year-old woman with one prior cesarean section presented with recurrent vaginal bleeding following an induced medical abortion, resulting in severe anemia with a hemoglobin level of 4.7 g/dL. Ultrasound revealed a 3.5 cm × 4 cm mass at the anterior isthmus consistent with cesarean scar ectopic pregnancy, which was managed by laparotomic wedge resection of the scar‑site lesion, bilateral uterine artery ligation, and transfusion of six units of whole blood. Patient 3: a 30 year-old woman (gravida 3, one prior cesarean delivery) had cesarean scar ectopic pregnancy missed during antenatal care; her diagnosis at 10 weeks followed the onset of pain and bleeding, and she underwent laparotomic resection of the scar‑site lesion. Histopathology confirmed cesarean scar ectopic pregnancy in all patients.

**Conclusion:**

Cesarean scar ectopic pregnancy is an emerging, potentially life-threatening complication in low-resource settings, where limited diagnostic capacity and low clinical suspicion often impede timely diagnosis and management. The avoidance of blind uterine evacuation until cesarean scar ectopic pregnancy is excluded, the adoption of lesion-tailored treatment strategies, and the strengthening of targeted clinical training and diagnostic access are critical to improve maternal health outcomes and reduce morbidity in these settings.

## Introduction

Cesarean scar pregnancy (CSP), also known as cesarean scar ectopic pregnancy (CSEP), is a rare type of ectopic pregnancy in which the pregnancy is implanted into a myometrial defect caused by a cesarean scar [1, 2]. It has an estimated incidence of approximately 1 in 2000 pregnancies, with a rising trend attributed to the global increase in cesarean delivery rates [1, 3]. However, reports from low-income settings remain limited, likely owing to underdiagnosis and underreporting [4]. The diagnosis of CSEP requires a high index of clinical suspicion and is primarily established through transvaginal ultrasound (TVUS) [5, 6]. If not identified promptly, CSEP can result in severe complications such as massive hemorrhage, uterine rupture, or progression to placenta accreta spectrum (PAS) [6]. In resource-limited settings, restricted access to skilled personnel and imaging technology presents substantial barriers to early diagnosis. Although delayed recognition of CSEP has been reported in prior studies [7, 8], this case series highlights severe maternal near-miss outcomes arising from such delays, underscoring the diagnostic and management challenges unique to resource-limited settings.

Moreover, the incidence of CSEP is anticipated to rise in low-income settings such as Ethiopia, where cesarean delivery rates are increasing, particularly in urban areas. Although CSEP remains a rare diagnosis, we encountered three cases over a 2-year period, January 2023 to May 2025, at a single teaching hospital, two of which resulted in maternal near-miss events owing to delayed diagnosis and definitive management. All patients included in this case series were of Ethiopian origin (East Africa). This case series outlines the diagnostic challenges, therapeutic interventions, and maternal outcomes observed, with the aim of informing clinical strategies for earlier recognition and improved management of CSEP in resource-constrained settings.

## Case description

### Patient 1

A 28-year-old Ethiopian woman, gravida 4, para 3, with a history of two prior cesarean deliveries, presented with intermittent vaginal bleeding of 2 weeks’ duration at a gestational age of 12 + 1 days, which was calculated from a reliable last menstrual period. She was referred from a primary hospital for further evaluation. The patient initially presented to a primary hospital with a 2-day history of vaginal bleeding. A diagnosis of threatened intrauterine miscarriage was made on the basis of clinical evaluation and obstetric ultrasound, which was misdiagnosed as an intrauterine pregnancy with a crown–rump length (CRL) corresponding to 10 weeks gestation. She was managed conservatively with pelvic rest for 2 weeks, and a follow-up ultrasound at the same facility revealed a viable intrauterine pregnancy with a CRL of 11 + 3 days and noted the presence of “retroplacental echogenic tissue,” as described by the referring institution. Owing to ongoing hemorrhage, she was transferred to a tertiary teaching hospital at 12 + 1 weeks for further assessment and management. She had no known preexisting medical conditions.

Two weeks following initial presentation at the referring center, the obstetrics and gynecology resident documented that the patient had passed some clotted blood and a lump of tissue. The initial vital signs were a blood pressure of 95/60 mmHg, pulse rate of 96 bpm, a respiratory rate of 20 breaths/minute, and a temperature of 37 °C. Physical examination revealed conjunctival pallor. The abdominal examination was unremarkable except for a well-healed midline subumbilical scar. On pelvic examination, there was minimal oozing from the cervix admitting one finger; bimanual evaluation revealed that the uterus was 10 weeks in size with no adnexal tenderness or masses. A transabdominal pelvic ultrasound revealed a 4.4 cm × 4 cm echogenic mass in the lower uterine segment. Laboratory studies revealed severe anemia (hemoglobin 7 g/dL), a platelet count of 432 × 10^3^/µL, and a white blood cell count of 9.6 × 10^3^/µL. On the basis of a working diagnosis of incomplete miscarriage, manual vacuum aspiration was attempted. However, the procedure was terminated when profuse hemorrhage ensued; the bleeding then ceased spontaneously without further intervention.

Repeat transabdominal ultrasound performed by a radiologist on the next day, revealed a 5.0 cm × 4.4 cm heterogeneously echogenic lesion occupying the endometrial cavity of the lower uterine segment. The mass contained internal hypoechoic components, significant Doppler flow, and evidence of myometrial invasion, with a thin rim of hypoechoic fluid outlining its superior margin. The radiologists’ differential diagnoses included gestational trophoblastic neoplasia and focal placental adherence at the previous cesarean scar.

Laboratory evaluation revealed a serum beta human chorionic gonadotropin (β-hCG) concentration of 22,000 mIU/mL and normal coagulation parameters (PT 11 s, aPTT 32 s, INR 1.1). With a diagnosis of a focally adherent retained product of conception, an obstetrician and gynecologist decided to proceed with medical management. She was transfused with two units of cross-matched blood, followed by a single intramuscular dose of methotrexate (50 mg/m^2^, total 90 mg). Serial β-hCG levels declined from 22,000 IU/L at baseline to 19,663 mIU/mL on day 3, 8199 mIU/mL on day 7, and 5658 mIU/mL on day 10. During this period, she experienced intermittent minimal vaginal bleeding but stable vital signs.

**Fig. 1:**
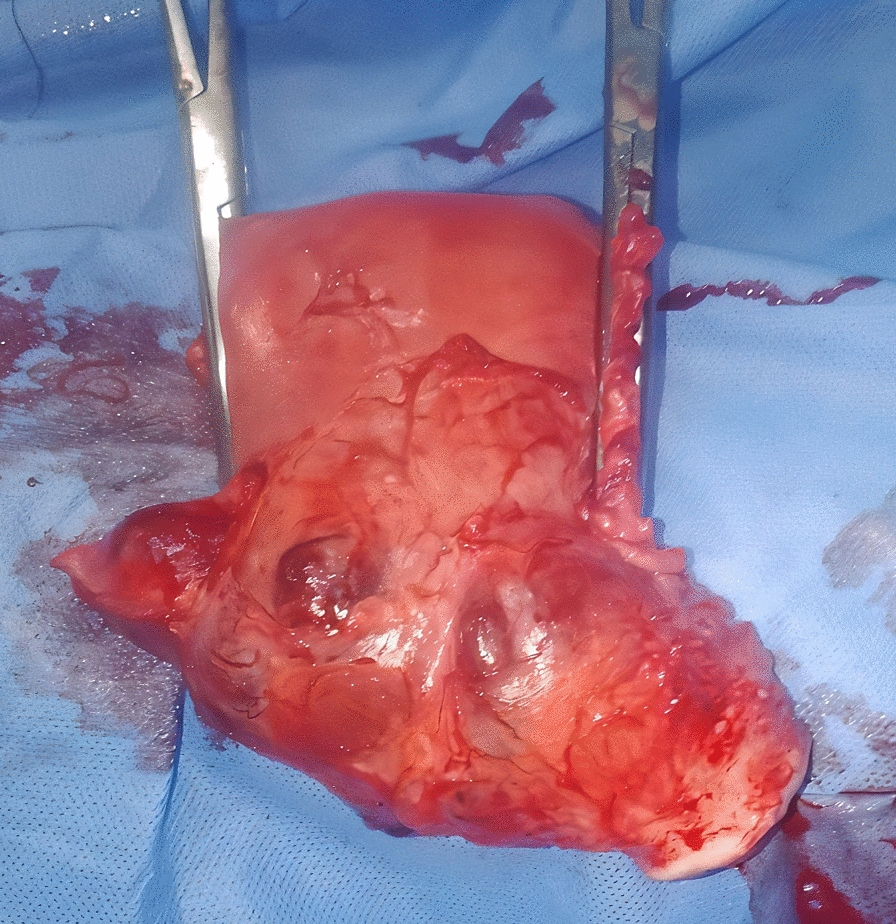
Post-hysterectomy gross specimen showing an irregular bluish bulge in the anterior lower uterine segment at the site of the prior cesarean scar, with marked vascular engorgement indicative of cesarean scar ectopic pregnancy

However, follow-up imaging revealed no reduction in lesion size. On day 12 post-methotrexate, she abruptly developed profuse vaginal bleeding. At that time, her blood pressure was 85/60 mmHg, her heart rate was 114 bpm, her respiratory rate was 24 breaths/minute, and her temperature was 36.8 °C. With a working diagnosis of actively bleeding cesarean scar ectopic pregnancy, the team proceeded with emergency laparotomy. Intraoperatively, the previous scar site was diffusely enlarged, with an irregular 5 cm × 4.4 cm bluish lesion and prominent vascular engorgement (Fig. 1). Owing to the size of the lesion, depth of invasion, and hemodynamic instability, a total abdominal hysterectomy was performed (Fig. 1).

**Fig. 2:**
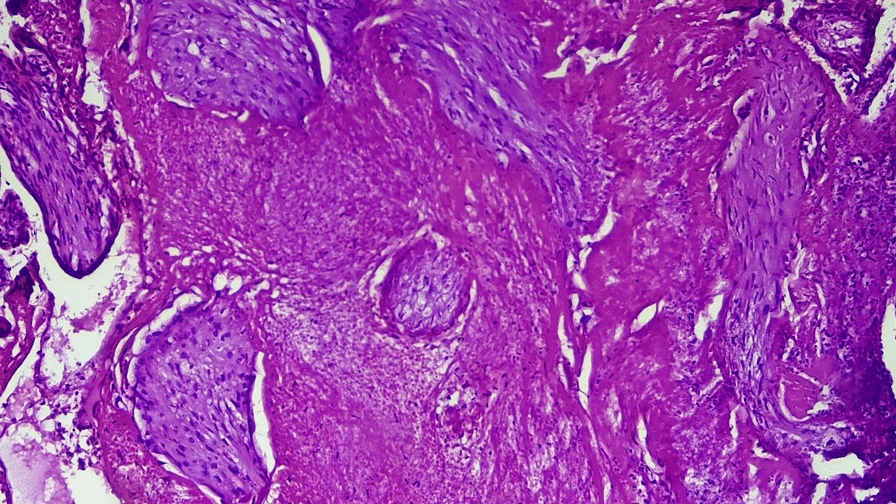
(Hematoxylin and eosin stain): medium-power photomicrograph demonstrating chorionic villi embedded within the myometrial muscle fibers

**Fig. 3:**
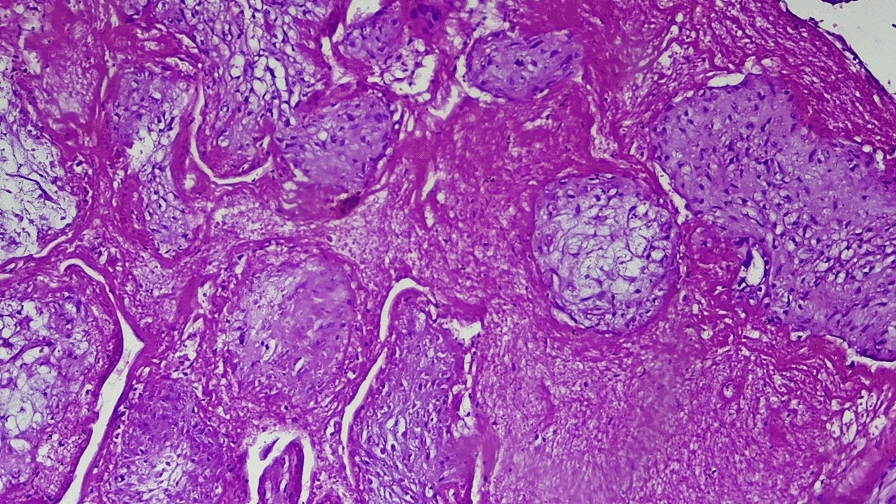
(Hematoxylin and eosin stain): medium-power microscopic examination reveals well-formed chorionic villi infiltrating through disorganized myometrial fibers

She received a total of five units of cross-matched whole blood. Postoperatively, she remained hemodynamically stable, was monitored in the recovery room, and was discharged on postoperative day 4. Histopathology confirmed that chorionic villi were embedded within the myometrium at the cesarean scar (Figs. 2 and 3). At her follow-up visit, she remained well, with a smooth postoperative course.

### Patient 2

A 25-year-old gravida 2 para 1 Ethiopian woman with one prior low transverse cesarean section presented to a primary hospital with a complaint of profuse vaginal bleeding of 1 week duration. Prior to her presentation, she had requested termination of an unplanned pregnancy and received 800 µg of misoprostol for a clinical diagnosis of a first trimester intrauterine pregnancy. She does not remember her last menstrual period and reports amenorrhea of approximately two and a half months’ duration. Over the next few days, she experienced intermittent vaginal bleeding with the passage of clots and small tissue fragments. However, her bleeding acutely worsened, prompting transfer from the primary hospital to a tertiary teaching hospital with a referral diagnosis of incomplete miscarriage. She had no known preexisting medical conditions.

On examination, she appeared pale with conjunctival pallor. Her vital signs were as follows: blood pressure 90/60 mmHg, pulse rate 108 bpm, respiratory rate 24/minute, and temperature 36.9 °C. The abdominal examination was unremarkable. Pelvic examination revealed blood-smeared vulva, a cervix admitting the tip of a finger, and a uterus estimated to be consistent with 8 weeks of gestation. Laboratory investigations revealed a hemoglobin level of 4.9 g/dL, a platelet count of 156,000/µL, and a white blood cell count of 7.6 × 10^3^/µL.

**Fig. 4:**
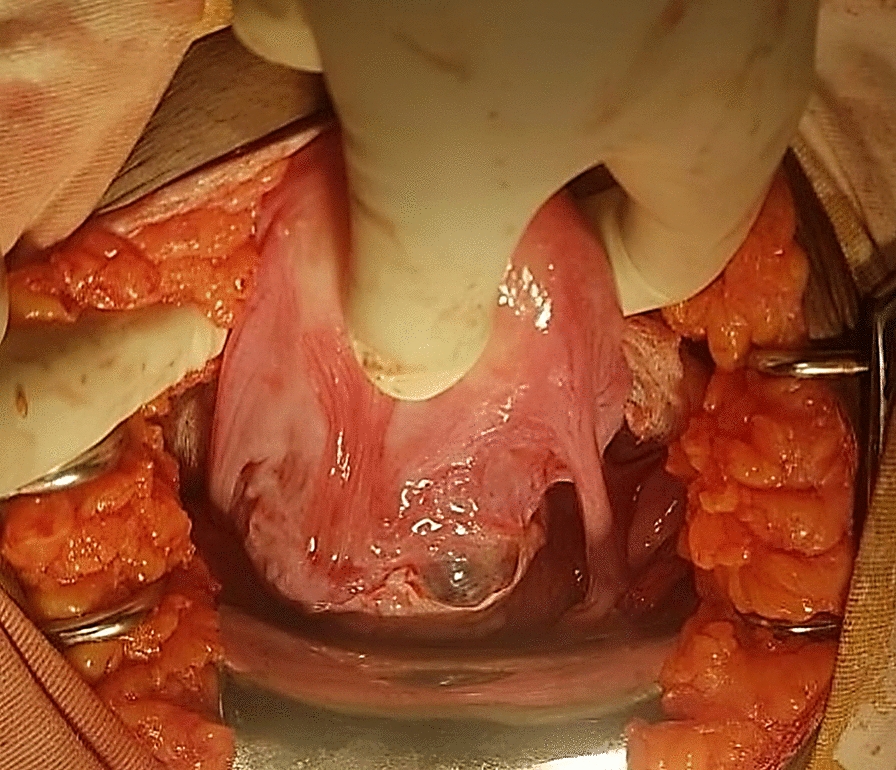
Intraoperative photo showing a 2.8 cm × 2.5 cm bluish mass, at a previous cesarean scar site covered by serosa

**Fig. 5:**
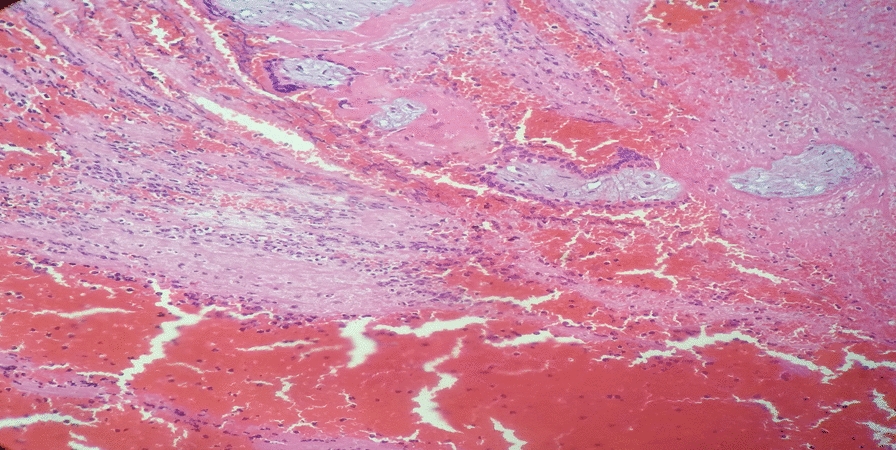
(Hematoxylin and eosin stain): low-power microscopic examination reveals variably sized chorionic villi embedded within a background of hemorrhagic and fibrinous stroma

**Fig. 6:**
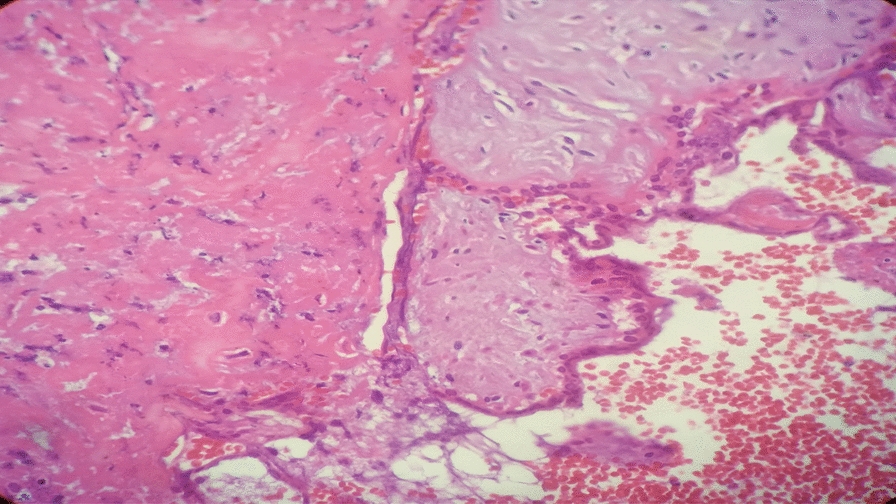
(Hematoxylin and eosin stain): medium-power histological examination revealed chorionic villi in close proximity to the fibrosed stroma

Transvaginal ultrasound revealed a uterus measuring 8.9 cm × 5 cm with a 4.5 cm × 4 cm echocomplex mass embedded within the isthmic region of the anterior uterine wall, demonstrating increased Doppler flow. The bladder wall appeared normal, the adnexa were unremarkable, and no free fluid was noted in the pelvis. On the basis of imaging and clinical findings, the attending obstetrician and gynecologist diagnosed the patient with a CSEP. After establishing this diagnosis, the team decided laparotomy as the appropriate management approach. Intraoperatively, a 3 cm × 2.5 cm bluish mass covered only by serosa was identified at the previous uterine scar (Fig. 4). Bilateral uterine artery ligation was performed, followed by wedge resection of the lesion and two-layer myometrial repair with 1-Vicryl suture. She was transfused with a total of six units of cross-matched whole blood. The patient was transferred to the recovery room in stable condition and discharged home on postoperative day 4 without complications during subsequent follow-up. Histopathology examination (H&E stain) revealed variably sized chorionic villi embedded within a background of hemorrhagic and fibrinous stroma, with some villi attached to densely fibrosed areas, which was consistent with abnormal implantation at a cesarean scar site (Figs. 5 and 6).

### Patient 3

A 30-year-old Ethiopian woman, gravida 3 para 1 miscarriage 1, with a history of one prior cesarean delivery, presented to a tertiary teaching hospital at a gestational age of 10 weeks and 1 day. She had initiated antenatal care earlier in pregnancy and was scheduled for routine follow-up. However, she developed lower abdominal pain and vaginal spotting and was subsequently referred from a private clinic with a diagnosis of CSEP, on the basis of ultrasound findings, for further evaluation and management. The patient had no known chronic medical conditions.

On examination, her vital signs were stable: blood pressure 110/70 mmHg, pulse rate 92 bpm, respiratory rate 22/minute, and temperature 36.7 °C. She had pink conjunctivae and nonicteric sclera. Abdominal examination revealed mild suprapubic tenderness. Pelvic examination revealed a closed cervix with minimal blood on the examining finger and no palpable adnexal mass.

Pelvic ultrasound revealed a uterus of normal size with a single, well-defined sac measuring 3.7 cm × 4.0 cm, located between the urinary bladder and anterior uterine wall. The sac was surrounded by a thick echogenic rim and had a vascular pedicle extending from the endometrial cavity through the previous cesarean scar. A yolk sac was visualized, but no embryonic pole was discernible. The mean sac diameter (MSD) was 2.04 cm, corresponding to a gestational age of 6 weeks and 4 days. The adnexa were unremarkable, and no free fluid was observed. Laboratory investigations revealed a white blood cell count of 6.25 × 10^9^/L, a hemoglobin level of 13.5 g/dL, a platelet count of 150,000/µL, and a serum β-hCG level of 3649 mIU/mL.

**Fig. 7:**
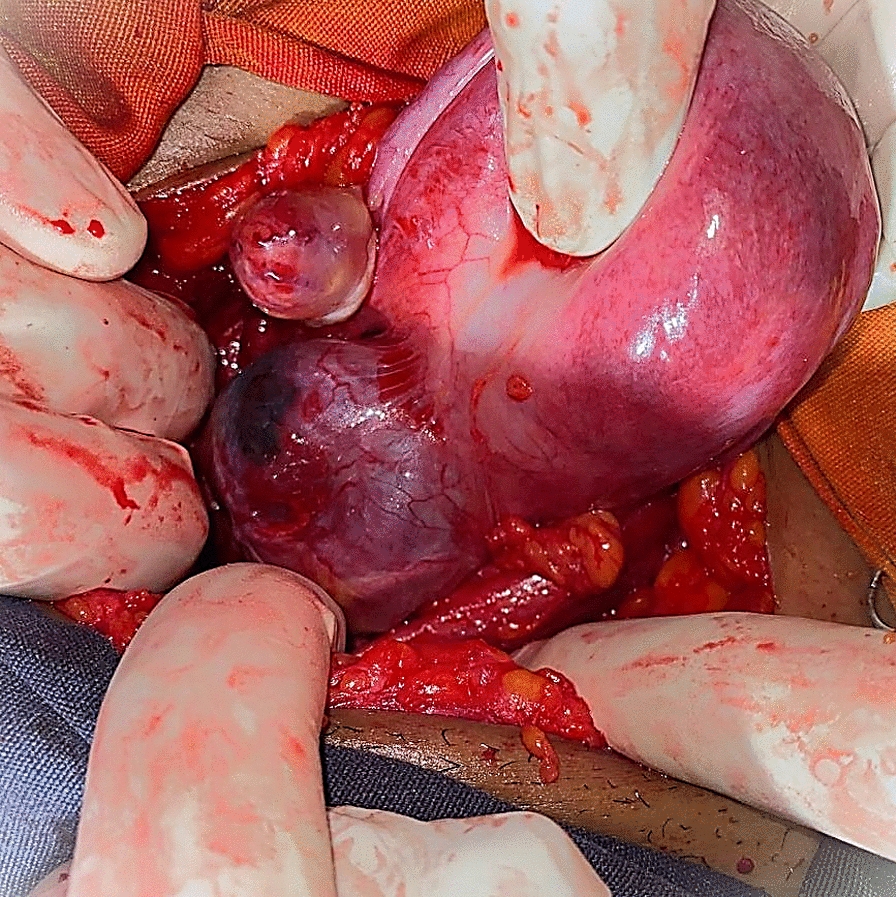
Intraoperative photograph showing a 5 cm × 4 cm bulging mass in the anterior lower uterine segment at the site of the previous cesarean section scar

**Fig. 8:**
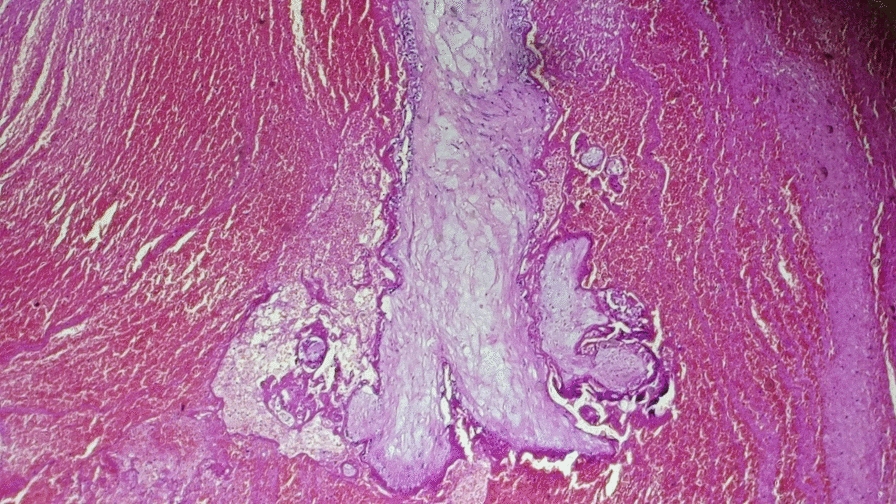
(Hematoxylin and eosin stain): low-power photomicrograph showing chorionic villi of varying sizes embedded within a markedly hemorrhagic stroma with focal hyalinization of the villi

**Fig. 9:**
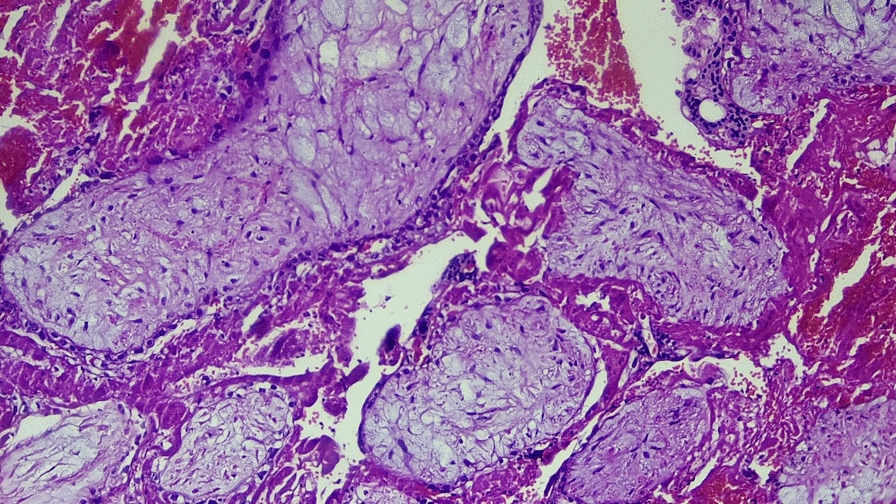
(Hematoxylin and eosin stain): medium-power photomicrograph demonstrating chorionic villi surrounded by fibrinous and fibrotic stroma

With a working diagnosis of cesarean scar ectopic pregnancy, the decision was made to proceed with laparotomy. Intraoperatively, a 5 cm × 4 cm bluish, bulging mass was identified at the site of the previous cesarean scar (Fig. 7). Wedge resection of the ectopic mass was performed, followed by uterine repair. The patient remained hemodynamically stable throughout the procedure and postoperative period. She was discharged in good condition, and at follow-up, her serum β-hCG levels decreased to undetectable levels. She experienced a smooth and uneventful recovery. Histopathologic examination (H&E staining) revealed chorionic villi of varying sizes dispersed within a background of hemorrhage and fibrinous connective tissue, which are characteristic of cesarean scar implantation (Figs. 8 and 9).

## Discussion

As the rate of cesarean sections continues to rise globally, CSEP has become increasingly prevalent, representing up to 8% of all ectopic pregnancies in some cohorts [9, 10]. However, data from low-income countries are lacking, and the true incidence is likely underdiagnosed and underreported [4]. To date, no published studies have addressed the burden of this condition in Ethiopia. This case series, reported from a tertiary teaching hospital in Ethiopia, highlights confirmed cases of CSEP with delayed diagnosis and underscores the need for increased awareness and diagnostic vigilance in similar settings.

CSEP occurs when a blastocyst is implanted into a deficient myometrial niche at the site of a previous cesarean delivery scar [1]. Retrospective analyses have identified independent predictors of CSEP, including the number of prior cesarean deliveries, advanced maternal age (≥ 35 years), gravidity exceeding three, and a history of multiple induced abortions [10–12]. In addition, specific studies have highlighted that a history of cesarean section performed in rural hospitals may further increase the risk of CSEP [11]. In our case, reported from a low-resource setting with most rural health care facilities, the context itself may represent an additional risk factor contributing to the increasing incidence of CSEP, reinforcing the notion that it is an emerging clinical challenge in such environments.

Patients with cesarean scar ectopic pregnancy most often present between 6 and 9 weeks of gestation and experience scant vaginal spotting and dull, crampy pain localized to the lower uterine segment, which is a subtler clinical picture than the sharp pain and heavier intraperitoneal bleeding observed with tubal ectopic pregnancies [6]. Published cohorts from high-income settings report an average gestational age at diagnosis of 7.5 weeks, with approximately 75% of patients experiencing vaginal bleeding and 60% reporting pelvic pain [6, 13]. In our series, patients 1 and 2 initially presented within this gestational window with mild, intermittent bleeding and dull, crampy lower abdominal pain, mirroring these documented patterns. However, diagnosis was delayed because of the low index of suspicion. This highlights the need for high clinical suspicion for scar implantation in any woman with a prior cesarean section presenting with early pregnancy bleeding and pain.

Transvaginal ultrasound is the cornerstone of cesarean scar ectopic pregnancy diagnosis, with classic sonographic features, including an empty uterine cavity and cervical canal, a gestational sac embedded in the anterior lower uterine segment at the scar niche, a thinned myometrial layer (< 3 mm) between the sac and bladder, and prominent peri-trophoblastic Doppler flow [6, 14]. However, after partial expulsion or blind evacuation attempts, retained products and hematomas can obscure these hallmarks, mimicking incomplete miscarriage and reducing TVUS sensitivity [14]. In our series, although confirmatory scans eventually revealed a niche-embedded sac with increased flow, initial misinterpretation led to delayed diagnosis in cases 1 and 2. In contrast, better recognition in patient 3, despite a relatively advanced gestational age, successfully averted critical morbidity.

Early diagnosis of CSEP is a critical determinant of clinical outcomes, requiring a high index of suspicion and careful transvaginal ultrasound evaluation [6]. However, in low-resource settings, diagnostic challenges such as limited access to imaging and trained personnel lead to delayed recognition [4]. Delayed diagnosis significantly increases the risk of life-threatening complications, including massive hemorrhage, uterine rupture, placenta accreta spectrum (PAS), and the potential need for emergency hysterectomy [6]. In our case series, the first patient was initially followed for 2 weeks with a diagnosis of threatened miscarriage, and then manual vacuum aspiration (MVA) was attempted for presumed incomplete miscarriage, which is a potential missed opportunity for early diagnosis. In the second patient, CSEP was diagnosed only after she developed anemia requiring blood transfusion. The third case represented a missed opportunity for diagnosis during routine antenatal care. Although the patient eventually benefited from timely surgical intervention once the condition was suspected, the initial oversight highlights how limited awareness and reliance on transabdominal rather than transvaginal ultrasound can delay recognition. Collectively, these findings emphasize the urgent need for a high index of suspicion and prioritizing transvaginal sonography (TVS) in low-resource settings where diagnostic delays are more common.

Two of the cases in this series met the World Health Organization (WHO) criteria for maternal near-miss events, defined as instances in which a woman nearly died but survived a life-threatening complication occurring during pregnancy, childbirth, or within 42 days of pregnancy termination [15]. The WHO identifies maternal near-miss cases via clinical, laboratory, and management-based criteria that indicate organ dysfunction [15]. In our case series, patient 1 required a hysterectomy owing to uncontrollable hemorrhage, and both patients 1 and 2 received transfusions of five units of blood—criteria that fulfill the WHO definition of maternal near misses. These cases identify CSEP as a concerning and potentially emerging driver of severe maternal morbidity and mortality in low-income regions, where diagnostic delays and inadequate access to advanced obstetric services exacerbate its impact.

Treatment options for cesarean scar ectopic pregnancy (CSEP) fall into medical, minimally invasive, and surgical categories tailored to the timing of diagnosis and the specific lesion type. Depending on individual factors, treatment may include medical therapy with systemic or local methotrexate and a range of surgical approaches, such as suction evacuation, dilation and curettage (D and C), uterine artery embolization, hysteroscopic or laparoscopic resection, and, when necessary, hysterectomy [16–19]. The current Society for Maternal–Fetal Medicine (SMFM) guidelines firmly discourage expectant or blind evacuation approaches [13]. Systemic methotrexate alone has a high failure rate (grade 1C), whereas ultrasound-guided local injection of methotrexate, often combined with prophylactic uterine artery embolization, can effectively treat small, well-vascularized CSEPs (grade 2C). For lesions larger than 3–4 cm, deeply myometrial-invasive, or associated with significant Doppler flow, the preferred strategy is operative resection via transvaginal, laparoscopic, or laparotomic wedge excision with meticulous myometrial repair (grade 1B) [13].

In our case series, case 1 illustrates how initial manual vacuum aspiration and systemic methotrexate led to delayed resolution, catastrophic hemorrhage, and ultimately hysterectomy, underscoring the dangers of non–scar-targeted therapy. In patient 2, delayed diagnosis culminated in severe anemia (hemoglobin, 4.9 g/dL), necessitating five units of blood transfusion before prompt surgical intervention; bilateral uterine artery ligation plus wedge resection arrested bleeding, preserved uterine integrity, and averted critical morbidity. In contrast, patient 3 benefited from early laparotomic excision at 10 weeks of gestation, which minimized blood loss and enabled rapid recovery.

In low-income settings such as Ethiopia, these cases highlight the urgent need for a high index of suspicion for cesarean scar implantation in any woman with a prior uterine scar presenting with first-trimester bleeding. Misinterpretation on routine transabdominal scans often delays diagnosis and increases the risk of severe morbidity, underscoring the importance of early, high-frequency transvaginal ultrasonography with color Doppler to detect hallmark features. Blind uterine evacuation must be strictly avoided until CSEP is definitively excluded, as non–scar-targeted interventions can precipitate catastrophic hemorrhage, profound anemia, massive transfusion, and emergency hysterectomy. Adopting lesion-tailored algorithms, local methotrexate for small implants, and prompt surgical resection with meticulous myometrial repair for larger or deeply invasive sacs can preserve fertility and reduce near-miss events.

With the rate of cesarean section steadily increasing, the public health system is expected to face a looming surge in cesarean-scar-related complications, particularly CSEP. Proactive measures such as restricting non-indicated cesareans, strengthening referral networks, fostering multidisciplinary teamwork, and instituting regular ultrasound training with case audits are essential to mitigate diagnostic delays, improve outcomes, and save lives.

### Strengths and limitations

A key strength of this case series is the documentation of three histologically confirmed cases of CSEP from Ethiopia, a low-resource setting where published data are extremely limited. The detailed clinical narratives, supported by intraoperative findings and histopathology, provide valuable lessons for clinicians and highlight the diagnostic pitfalls that can lead to maternal near-miss events. The inclusion of patient perspectives further enriches the report by underscoring the emotional and psychosocial impact of delayed diagnosis.

Nevertheless, the small number of cases limits the generalizability of the findings. As with most case series, the observations cannot establish incidence or causality, and the retrospective nature of data collection may have introduced reporting bias. The absence of advanced imaging modalities such as magnetic resonance imaging (MRI) also restricted the ability to fully characterize lesion morphology and vascular involvement, which may have provided additional diagnostic clarity. Moreover, against global standards, some management decisions warrant caution: methotrexate use in patient 1 for a mass > 4 cm is debatable, and transfusion protocols recommend adding fresh frozen plasma after ≥ 4 units of red cells. Variations in management, such as immediate laparotomy for patient 3 with low hCG and minimal bleeding versus initial medical management for patient 1 with higher hCG and heavier bleeding, reflect contextual judgment rather than strict adherence to standardized protocols.

## Conclusion

This case series highlights CSEP as an emerging and potentially life-threatening complication in low-resource settings, where delayed presentation, limited diagnostic capacity, and low clinical suspicion frequently hinder timely recognition and appropriate management. Such delays substantially increase the risk of severe maternal morbidity, including maternal near-miss events, as demonstrated in two of our cases. Blind uterine evacuation should be strictly avoided until CSEP is excluded, and lesion-tailored management strategies should be prioritized to preserve fertility and reduce morbidity. With cesarean delivery rates steadily rising, the incidence of CSEP is expected to increase, increasing its public health impact. Strengthening targeted clinical training, expanding access to high-quality imaging, improving referral pathways, and promoting judicious use of cesarean delivery are essential measures to mitigate diagnostic delays, optimize management, and save lives in similar low-resource environments.

## Data Availability

Data and materials are available when the corresponding author is contacted.

## Abbreviations

β-hCG: Beta human chorionic gonadotropin
CSEP: Cesarean scar ectopic pregnancy
CSP: Cesarean scar pregnancy
D and C: Dilation and curettage
Hb: Hemoglobin
MVA: Manual vacuum aspiration
PAS: Placenta accreta spectrum
SMFM: Society for Maternal–Fetal Medicine
TVS: Transvaginal sonography
WHO: World Health Organization
